# Serum GFAP as a biomarker for disease severity in multiple sclerosis

**DOI:** 10.1038/s41598-018-33158-8

**Published:** 2018-10-04

**Authors:** A. Abdelhak, A. Huss, J. Kassubek, H. Tumani, M. Otto

**Affiliations:** 1 Department of Neurology, University Hospital of Ulm, Ulm, Germany; 2 Department of Experimental Neurology, University of Ulm, Ulm, Germany; 3 Speciality Clinic of Neurology Dietenbronn, Schwendi, Germany

**Keywords:** Multiple sclerosis, Neurodegenerative diseases

## Abstract

While neurofilament light chain (NfL) measurement in serum is a well-established marker of neuroaxonal damage in multiple sclerosis (MS), data on astroglial markers in serum are missing. In our study, glial fibrillary acid protein (GFAP) and NfL were measured in cerebrospinal fluid (CSF) and serum of MS patients and patients with other non-inflammatory neurological diseases (OND) using the Simoa technology. Clinical data like age, gender, expanded disability status scale (EDSS) and MRI findings were correlated to neurochemical markers. We included 80 MS patients: 42 relapsing-remitting MS (RRMS), 38 progressive MS (PMS), as well as 20 OND. Serum GFAP levels were higher in PMS compared to RRMS and OND (p < 0.001, p = 0.02 respectively). Serum GFAP levels correlated with disease severity in the whole MS group and PMS (Spearman-rho = 0.5, p < 0.001 in both groups). Serum GFAP correlated with serum NfL in PMS patients (Spearman-rho = 0.4, p = 0.01). Levels of serum GFAP were higher with increasing MRI-lesion count (p = 0.01). in summary, we report elevated levels of GFAP in the serum of MS patients. Since serum levels of GFAP correlate with the clinical severity scores and MRI lesion count, especially in PMS patients, it might be a suitable disease progression marker.

## Introduction

The role of astrocytes in multiple sclerosis (MS) has been investigated extensively. Beyond their role in scar formation, astrocytes are a potent secretor of different proinflammatory cytokines and cytotoxic factors and contribute to mitochondrial dysfunction^1,2^. Astrocyte subtype A1 is a potent direct killer of the neurons and oligodendrocytes^3^. Glial fibrillary acidic protein (GFAP) is a well-established marker of astrogliosis as numerous studies described its use for MS and reported correlations with disease severity, the extent of neuroinflammation and progression^4–8^. Using a proteomic approach, we previously suggested the use of GFAP in subtyping progressive forms of MS^9^. However, up to now, data regarding levels of GFAP in blood samples of MS patients are scarce due to the detection limit of available assays^10^. However, with the introduction of the highly sensitive immunoassay platforms, this now is possible as shown after traumatic brain injury^11^. In our study, we investigated the diagnostic use of blood levels of GFAP in relation to the neuroaxonal damage marker, neurofilament light chain (NfL), and the clinical subtype of MS.

## Results

### Patient characteristics

Paired serum and cerebrospinal fluid (CSF) samples of 80 MS patients and 20 other non-inflammatory neurological diseases (OND) patients were analysed. Four relapsing-remitting MS between relapses (RRMS-) patients received disease-modifying therapy (DMT); two received interferon-1-β (INF-β), one received Natalizumab, and another one was on Alemtuzumab. Three secondary progressive MS (SPMS) patients were on DMT (Natalizumab, INF-β and Fingolimod). Teriflunomide was used in one RRMS patient during acute exacerbation (RRMS+) and Natalizumab in another patient. A cranial magnetic resonance imaging (MRI) at the time of lumbar puncture (LP) was available from 64 MS patients. For 59 MS patients, additional MRIs of the cervical and thoracic cord were available. All clinical characteristics of the patients along with serum and cerebrospinal fluid (CSF) levels of GFAP and NfL are summarised in Table 1. All the below mentioned clinical and laboratory parameters did not differ between the treatment-naive patients and patients under DMT.

**Table 1:** Clinical characteristics and biomarker levels in CSF and serum of the included subjects. All given values are median values with the interquartile range in brackets. (25–75 percentile).

median (25–75 percentile)	Multiple sclerosis (*n* = 80; 46♀ and 34♂)				Controls (*n* = *20*; 13 ♀ and 7 ♂
	RRMS− *(n* = *24)3)*	RRMS+ (*n* = *18)*	SPMS (*n* = *13)*	PPMS (*n* = *25)*	
Age	36 (27–49)	28 (24–43)	50 (49–58)	53 (45–59)	44 (27–52)
EDSS	2.25 (1.5–4.75)	2.0 (1.5–2.0)	6.5 (6.0–7.5)	4.5 (3.0–6.5)	n/a
MSSS	5.9 (2.9–7.8)	5.6 (2.9–6.3)	7.5 (6.5–8)	6.8 (5.9–8.6)	n/a
ARMSS	5.9 (2.2–7.4)	4.6 (3.4–5.9)	8.7 (7.5–8.9)	6.8 (3.3–7.9)	n/a
Disease duration (in months)	61 (6–86)	6 (1–15)	294 (276–420)	60 (40–216)	n/a
Albumin quotient (Q_alb_)	3.5 (0.5–5.5)	5.0 (3.4–5.8)	4.2 (3.0–5.7)	3.8(1.7–6.5)	1.0 (0.6–1.3)
CSF GFAP in pg/ml	6925 (4365–10600)	7935.2 (5480.7–11310.4)	12329.8 (8038.9–16036.1)	10300 (7100–13680)	6157.8 (2452.5–7967.5)
Serum GFAP in pg/ml	113.5 (78.3–137.8)	78.2 (55.0–165.0)	145.9 (86.5–245.9)	130.5 (98–167.5)	92.33 (59–139.1)
CSF NfL in pg/ml	1718.7 (998.9–3347.5)	1674.0 (824.0–3210.0)	1570.0 (1450.0–2491.6)	1241 (898.0–2240.0)	584.5 (371.5–827.1)
Serum NfL in pg/ml	13.9 (7.9–27.3)	15.0 (11.0–24.0)	28.6 (19.7–44.4)	17.4 (10.8–22.4)	9.2 (6.0–12.2)

n = number, ♀ = female, ♂ = male, CSF: cerebrospinal fluid, RRMS-: relapsing-remitting multiple sclerosis between relapse, RRMS + : relapsing-remitting multiple sclerosis during acute exacerbation, SPMS: secondary progressive multiple sclerosis, PPMS: primary progressive multiple sclerosis, n/a: not applicable. EDSS: expanded disability score scale, MSSS: multiple sclerosis severity score, ARMSS: age-related multiple sclerosis severity score.

73.4% (*n* = 47) of the MS patients had >9 lesions and 25% (*n* = 16) between 2–9 lesions; only one MS patient had less than two lesions in the cranial MRI. Both supra- and infratentorial lesions were the most common MRI lesion pattern and were found in 57.8% (*n* = 37) followed by supratentorial lesions alone (40.6%, *n* = 26). Gd+ and Gd- lesions were equally distributed (50% each, n = 32) and spinal lesions in 66.1% (*n* = 39) of all available scans.

### GFAP

GFAP in CSF and serum showed strong correlation in MS patients (*Spearman*’*s rho* = 0.6, p < 0.001) and in controls (*Spearman’s rho* = 0.7, p < 0.001).

In MS patients CSF and serum GFAP increased with age (*Spearman rho* = 0.4, p < 0.001 for both). Stronger correlations were found in the controls (*Spearman rho* = 0.6 and 0.8 p < 0.001 respectively). Moreover, CSF, but not serum GFAP, correlated with albumin quotient (Q_alb_) in relapsing and progressive MS patients (*Spearman rho* = 0.3 and 0.4 respectively, p = 0.02 for both). Gender did not affect GFAP levels neither in CSF nor in serum.

**Figure 1:**
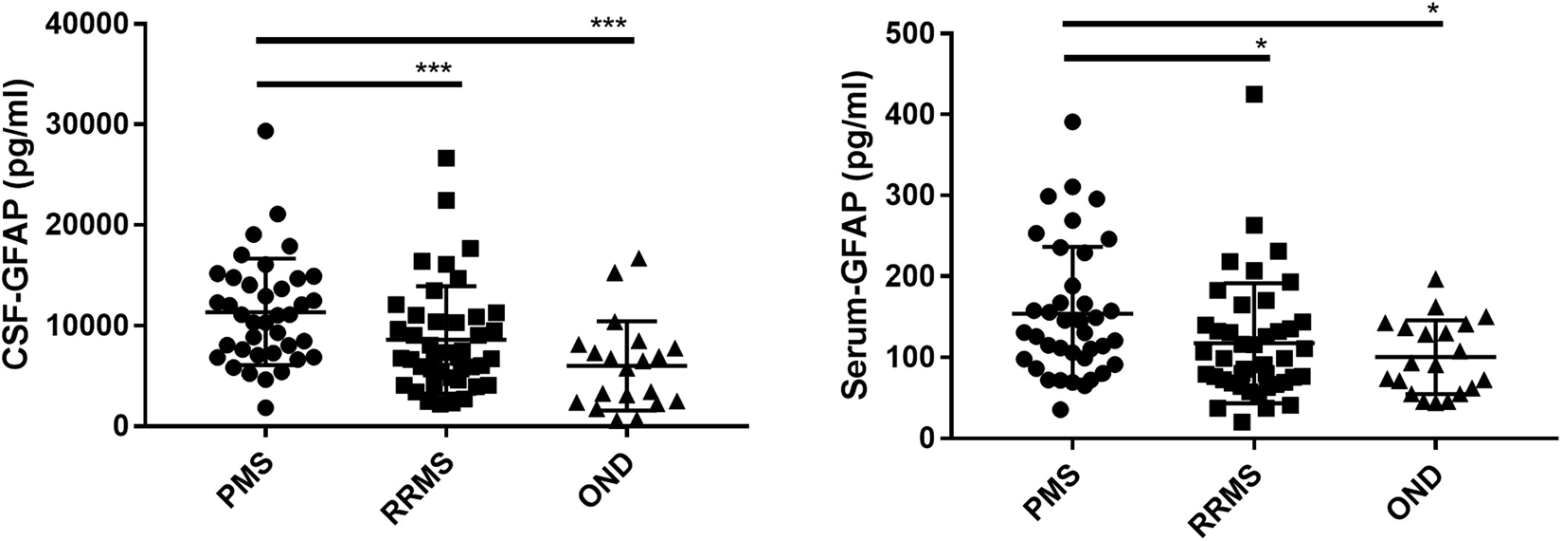
GFAP CSF and serum levels in multiple sclerosis patients and patients with other non-inflammatory neurological diseases (OND). GFAP: glial fibrillary acidic protein. CSF: cerebrospinal fluid, PMS: progressive multiple sclerosis, RRMS: relapsing-remitting multiple sclerosis. P-values were calculated with Kruskal-Wallis test followed by Dunn’s multiple comparison tests. ^*^p < 0.05; ^***^p < 0.001.

CSF and serum GFAP levels were higher in patients with progressive MS (PMS) compared to RRMS (p = 0.001 and 0.02 respectively) and controls (p = 0.001 and 0.01 respectively) (Fig. 1). However, after correction for age, the difference between PMS and RRMS was no longer statistically significant. Similarly, the correlation between CSF and serum GFAP with disease duration (0.3 and 0.4 respectively, p < 0.01) turned insignificant after correction for age. The concentration of GFAP in CSF and serum in RRMS did not differ significantly from the controls (p = 0.05 and 0.5). Correlations between CSF and serum GFAP and different clinical outcome parameters are reported in Table 2.

**Table 2:** Correlation matrices in different subgroups.

	Whole MS group (*n* = *80*)			RRMS (*n* = *42*)			PMS (*n* = *38*)		
	EDSS	MSSS	ARMSS	EDSS	MSSS	ARMSS	EDSS	MSSS	ARMSS
GFAP CSF	0.4^**^	n.s	n.s	n.s	n.s	n.s	n.s	n.s	n.s
GFAP serum	0.5^***^	0.4^**^	0.3^*^	0.4^*^	n.s	n.s	0.5^***^	n.s	0.4^**^
NfL CSF	n.s	n.s	n.s	n.s	n.s	n.s	n.s	n.s	n.s
NfL serum	0.3 ^**^	n.s	n.s	n.s	n.s	n.s	0.3^*^	n.s	n.s

RRMS: all relapsing-remitting MS patients (RRMS- and RRMS+); PMS: all patients with progressive MS (SPMS and PPMS); values are Spearman r, ^*^p-value < 0.05; ^**^p-value < 0.01; ^***^p-value < 0.001. EDSS: expanded disability score scale, MSSS: multiple sclerosis severity score, ARMSS: age-related multiple sclerosis severity score.

**Figure 2:**
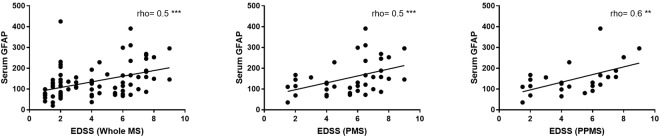
Spearman correlation (rho) between serum glial fibrillary acidic protein (GFAP) and expanded disability status scale (EDSS) in the whole multiple sclerosis (MS), progressive (PMS) and primary progressive (PPMS) patients. ^**^p < 0.01, ^***^p < 0.001.

Only in primary progressive MS (PPMS), serum GFAP correlated strongly with expanded disability status scale (EDSS) (*Spearman rho* = 0.6, p = 0.002) and with age-related multiple sclerosis severity score (ARMSS) (*spearman rho* = 0.4, p = 0.04) (Fig. 2). In PMS and PPMS but not RRMS patients, the correlation between serum GFAP and EDSS remains significant after correction for age (*Spearman rho* = 0.4 and 0.5, p = 0.008 and 0.02 respectively).

### NfL

CSF and serum levels of NfL showed a high correlation in the MS patients and controls (*Spearman rho* = 0.7 and 0.6, p < 0.001 and = 0.008, respectively). In contrary to GFAP, NfL levels did not correlate with age or Q_alb_, except for a weak correlation between serum NfL and age (*Spearman rho* = 0.2, p = 0.048). Serum NfL correlated with disease duration (*Spearman rho* = 0.4, p < 0.001), but not after correction for age.

**Figure 3:**
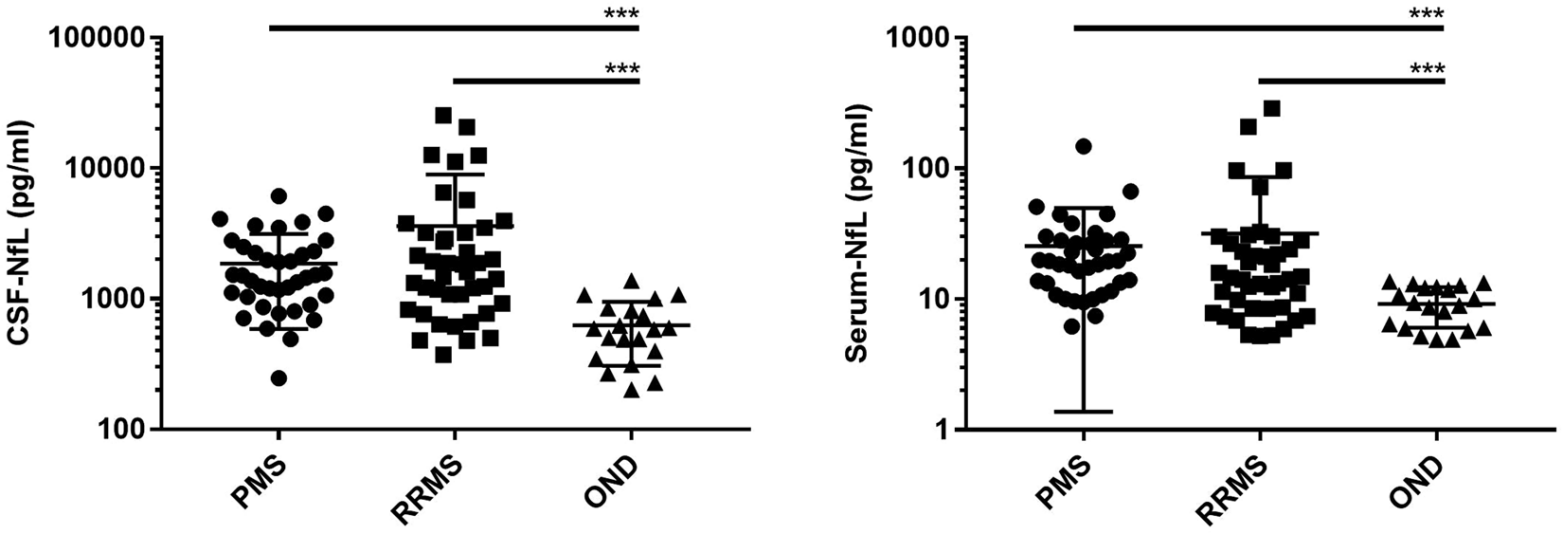
NfL CSF and serum levels in multiple sclerosis patients and patients with other non-inflammatory neurological diseases (OND). NfL: neurofilament light chain, CSF: cerebrospinal fluid, PMS: progressive multiple sclerosis, RRMS: relapsing-remitting multiple sclerosis. OND: other non-inflammatory neurological diseases. P-values were calculated with Kruskal-Wallis test followed by Dunn*’*s multiple comparison test. ^***^p < 0.001.

Concentrations of NfL in CSF and serum were higher in PMS and RRMS (p < 0.001 for all comparisons) compared to controls. However, NfL in CSF and serum did not differ between progressive and relapsing MS course (Fig. 3). Correlations with the different clinical parameters are listed in Table 2.

### GFAP in correlation to NfL

**Figure 4:**
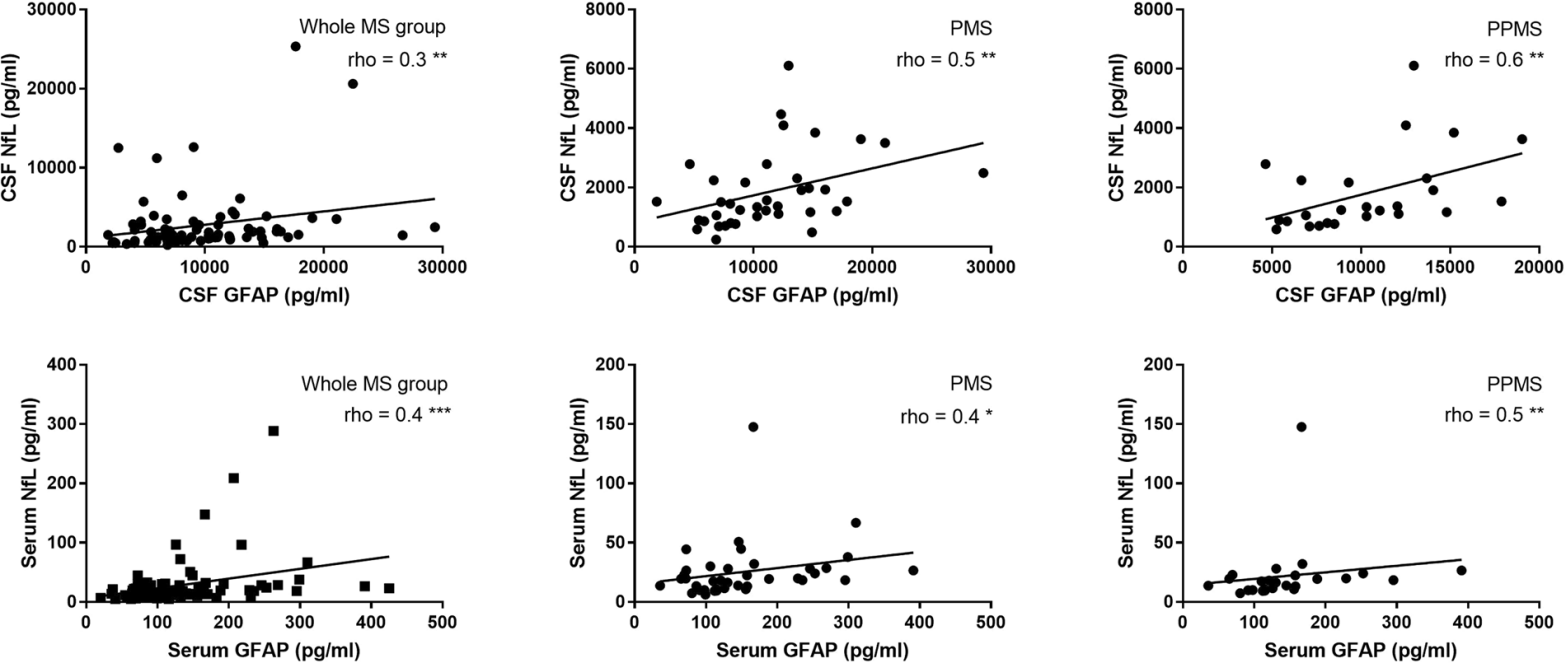
Spearman correlation (rho) between glial fibrillary acidic protein (GFAP) and neurofilaments light (NfL) in cerebrospinal fluid (CSF) and serum in the whole multiple sclerosis (MS), progressive (PMS) and primary progressive (PPMS) patients. ^*^p < 0.05, ^**^p < 0.01, ^***^p < 0.001.

In the whole MS group and in PMS patients, GFAP correlated with NfL in CSF (*Spearman rho* = 0.3 and 0.5, p = 0.007 and 0.003, respectively) and serum (*Spearman rho* = 0.4 for both, p < 0.001 and = 0.01, respectively). The correlation was even stronger in PPMS (*Spearman rho* = 0.6, p = 0.003 for CSF and 0.5, p = 0.007 for serum values) (Fig. 4). In RRMS patients, only serum GFAP and NfL showed a significant correlation (*Spearman rho* = 0.4, p = 0.01).

**Figure 5:**
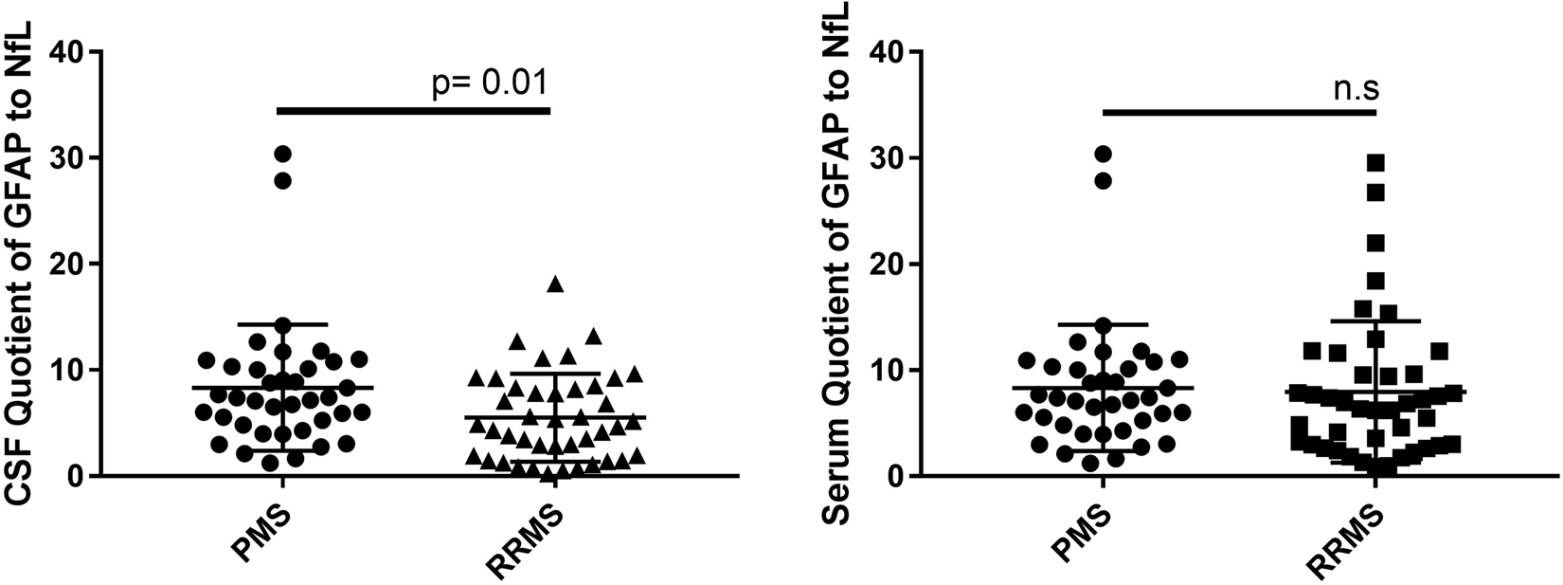
Quotient of glial fibrillary acidic protein (GFAP) and neurofilaments light (NfL) in cerebrospinal fluid (CSF) and serum in progressive multiple sclerosis patients (PMS) compared to relapsing-remitting multiple sclerosis (RRMS). n.s.: not significant.

To describe the relation of astroglial impact versus axonal impairment, we introduced a quotient of GFAP to NfL in CSF. This ratio (Q-GFAP/NfL-CSF) yielded significantly higher values for PMS patients (median: 7.3, 25–75 percentile: 4.8–10.3) compared to RRMS patients (median: 4.8, 25–75 percentile: 1.9–8.3) (p < 0.01) (Fig. 5).

### Imaging correlations

CSF and serum concentration of GFAP was higher in patients with >9 lesions compared to patients with 2–9 lesions (2028 vs 1520 and 133 vs 80.5, p = 0.01 for both). GFAP in CSF and serum were not influenced by the location of the lesions, the presence of gadolinium enhancement (Gd+) or spinal lesions. We found no correlation between NfL in CSF and serum with the MRI parameters.

## Discussion

In contrast to the well-established neuroaxonal marker NfL^12^, data regarding the concentration of astroglial markers in serum are still scarce. This is especially important as there is mounting evidence of an altered astroglial response in MS^9,13^. In our study, we report elevated levels of GFAP in CSF and serum in MS patients using the SIMOA assay.

Our results from CSF are in agreement with earlier studies using the traditional ELISA^4–7,13–19^. We report a strong correlation between GFAP in CSF and serum in MS patients as well as controls, as described previously for NfL in CSF and serum using the same method^20^. The positive correlation between GFAP and age may be explained by the well-described increasing GFAP expression from astrocytes with ageing, which may be mediated through hormonal mechanisms^21^.

We describe higher levels of serum and CSF GFAP in progressive MS types, but not necessarily in RRMS patients, compared to controls, and their correlation with disease severity. This may emphasise an increasing role of astrogliosis in advanced stages of the disease^9,22,23^ but not in the early phase. The observed strong correlation between the astroglial marker GFAP with a neuroaxonal damage marker like NfL in CSF and serum in PMS substantiate this assumption. Moreover, the chronic active and inactive lesions dominate in the brain of PMS patients^24^ with abundant scare forming astrocytes, which are known to inhibit remyelination and axonal regeneration^1,25^. On the other hand, the reactive astrocytes (RA) found in the predominantly active lesions in RRMS play a dual role; they may increase inflammatory activity by secreting proinflammatory cytokines, cytotoxic factors and recruiting various subtypes of immune cells. However, they may terminate the immune response, support differentiation of regulatory T-Helper cells and promote remyelination^1^. This dual role of RA may explain the absence of correlation between GFAP and EDSS in RRMS patients.

Beyond the degree of astrogliosis, the different lesion patterns in MS brains vary in the extent of neuroaxonal demise; active lesions exhibit more axonal damage than chronic active or inactive lesions. Hence, a ratio of GFAP to NfL might reflect, at least partially, the predominant lesion pattern and differentiate between PMS to RRMS. The higher values found in PMS patients may be explained by the predominance of the chronic lesions in comparison to RRMS patients.

Interestingly, the correlation of GFAP with the different clinical parameters seems particularly strong and constant in PPMS. Thereupon, it would be interesting to explore a possible role of serum GFAP as a marker of disease severity in patients with PPMS.

Moreover, in our study, the correlation of GFAP with the clinical severity scores was most prominent in serum. GFAP expression occurs mainly on the branches of astrocytes, which also participate in the formation of blood-brain barriers and therefore are in direct contact with the blood vessels^26,27^. Sofroniew and Vinters *et al*. reported increased GFAP expression on the astrocyte processes and endfeet in response to inflammation in mice with experimental autoimmune encephalomyelitis^26^. Taking into consideration the perivascular distribution of most of MS lesions^28,29^, GFAP might partially drain directly into the blood and not into the CSF.

Nevertheless, caution is warranted while interpreting the meaning of GFAP levels as both neurotoxic A1 and the possibly neuroprotective A2 reactive astrocytes overexpress GFAP^30^. To the best of our knowledge, while the role of A1 astrocytes is established in neuroinflammation, the presence and role of A2 astrocytes are still to be defined^3^.

Beyond that, the observed higher CSF and serum NfL concentrations in PMS and RRMS underscore their value as a marker of neuroaxonal demise as reported in various studies^18,19,31^.

Our findings still have to be confirmed in further prospective studies with larger sample size, follow up samples to explore the temporal evolution of the mentioned biomarkers during and after acute exacerbations, and more detailed MRI protocols addressing further aspects beyond inflammatory lesion load/volumentry such as white and grey matter atrophy and cortical pathology. However, the novelty of the method and the present results might give new insights into the pathophysiology of the progressive MS forms.

## Conclusions

In summary, we report elevated levels of GFAP in the serum of MS patients. Correlations between GFAP and NfL, as well as disease severity, might indicate a possible role of astrocytes in the neuroaxonal demise in MS. Since serum levels of GFAP correlate with the EDSS, especially in PPMS patients, it might be a suitable disease severity marker that is easily accessible for follow-up observations. Certainly, further studies are recommended to validate these findings.

## Methods

### Patient selection

In a prospective study, CSF and serum samples were collected from eighty MS patients attending the department of neurology at the University of Ulm between 2012 and 2017 as a part of the routine diagnostic workup and were included into the biomaterial bank using rigid standard operating procedures (SOPs). In all patients, the diagnosis was revised according to the 2017 revision of the McDonald criteria^32^. Patients were divided into relapsing-remitting MS, with the LP and serum sampling performed during acute exacerbation (RRMS+), or afterword (RRMS-), PPMS and SPMS. Exacerbations were defined as focal neurological disturbance lasting more than 24 h, without an alternate explanation^33^. In RRMS+ patients, CSF and serum samples collection were performed before the initiation of methylprednisolone treatment. The clinical severity was measured by assessing the EDSS, multiple sclerosis severity score (MSSS) as well as the ARMSS as reported recently^34^. The control group consisted of eighteen patients with other non-inflammatory neurological diseases (OND) with normal CSF and magnetic resonance imaging MRI findings (e.g., hypesthesia, dissociative disorder and tension headache).

### CSF and serum sampling

CSF and serum samples were taken on the day of presentation and stored according to consensus protocol for the standardisation of CSF collection and biobanking^35^. Haemolytic CSF specimens were excluded. LP in RRMS+ patients was performed within the first three months after the onset of the exacerbation. Only in three out of 18 RRMS+ -patients, the treatment was initiated after a long latency (42, 60 and 90 days), while the majority of these patients was treated in less than 2 Weeks. We used the CSF-serum quotient for albumin (Q_alb_)^36^ as an indicator for the blood-CSF barrier (BCB) function.

### GFAP and NfL measurements

Both, GFAP and NfL were measured in CSF and serum using the Simoa technology and GFAP Discovery and NfL Early Access assays (Quanterix Corporation, Lexington, MA, USA). Samples were diluted, as recommended by the manufacturer, and concentrations were calculated using the corresponding standard curve. The intra-assay coefficient of variation (CV) was assessed by measuring a QC serum, and CSF sample in 5 replicates and a CV below 10% was obtained. Only samples that showed a higher signal than the lowest point of the standard curve (1.37 pg/ml for GFAP and 0.69 pg/ml for NfL) were taken into account for the analysis (this was true for all CSF and serum samples).

### Magnetic resonance imaging (MRI) scans

MRI scans of the brain and spinal cord were performed on a 1.5 Tesla MRI scanner (Symphony, Siemens, Erlangen, Germany) according to a standard protocol including T1-weighted spin-echo (SE) axial scans with and without application of gadolinium-DTPA (Gd) as well as T2-weighted turbo inversion recovery with frequency-selective fat saturation (FLAIR) coronal scans, where the hyperintense lesions larger than three mm^2^ were quantified. Scan results were divided into subgroups according to the number of lesions (≤1, 2–9, and >9 lesions), localisation (supra- vs infratentorial) and Gadolinium (Gd) enhancement (Gd+ vs Gd−). The scans were analysed by a rater blinded to the CSF and serum analysis results.

### Statistical methods

All statistical tests were performed using SPSS® Statistics version 25 (IBM Corporation, Armonk, NY, USA). The Shapiro-Wilk test was used to examine the distribution of the data. Mann-Whitney U-test was used to compare medians in skewed distributed parameters. A general linear model was applied to account for a possible confounding bias caused by the strong correlation between GFAP levels and age. The Spearman’s rho test was used to measure correlation. A p-value ≤ 0.05 was considered as statistically significant. Figures were made using GraphPad Prism 6 software (GraphPad Software Inc., La Jolla, CA, USA).

### Ethical approval

The study was reviewed by the ethics committee of the University of Ulm and all experimental protocols were approved (approval number 20/10). Our study was performed in accordance with the ethical standards the 1964 Declaration of Helsinki. Written informed consent for the study participation was obtained from all patients participating in this study.

## Data Availability

The datasets generated during and/or analysed during the current study are available from the corresponding author on reasonable request.
