# Author Correction: Serum GFAP as a biomarker for disease severity in multiple sclerosis

**DOI:** 10.1038/s41598-019-43990-1

**Published:** 2019-06-05

**Authors:** A. Abdelhak, A. Huss, J. Kassubek, H. Tumani, M. Otto

**Affiliations:** 1 Department of Neurology, University Hospital of Ulm, Ulm, Germany; 2 Department of Experimental Neurology, University of Ulm, Ulm, Germany; 3 Speciality Clinic of Neurology Dietenbronn, Schwendi, Germany

Correction to: *Scientific Reports* 10.1038/s41598-018-33158-8, published online 04 October 2018

In Figure 5, the values for the PMS subgroup are duplicated in both 5a and 5b. The correct Figure 5 appears below as Figure 1.

**Figure 1:**
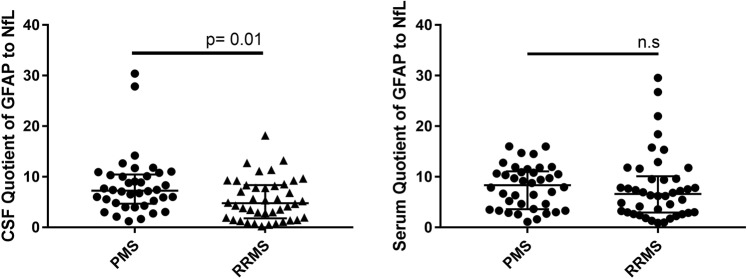
Quotient of glial fibrillary acidic protein (GFAP) and neurofilaments light (NfL) in cerebrospinal fluid (CSF) and serum in progressive multiple sclerosis patients (PMS) compared to relapsing-remitting multiple sclerosis (RRMS). n.s.: not significant.

